# Cannabidiol Overcomes Oxaliplatin Resistance by Enhancing NOS3- and SOD2-Induced Autophagy in Human Colorectal Cancer Cells

**DOI:** 10.3390/cancers11060781

**Published:** 2019-06-05

**Authors:** Soyeon Jeong, Bu Gyeom Kim, Dae Yeong Kim, Bo Ram Kim, Jung Lim Kim, Seong Hye Park, Yoo Jin Na, Min Jee Jo, Hye Kyeong Yun, Yoon A. Jeong, Hong Jun Kim, Sun Il Lee, Han Do Kim, Dae Hyun Kim, Sang Cheul Oh, Dae-Hee Lee

**Affiliations:** 1 Division of Oncology/Hematology, Department of Internal Medicine, Korea University Guro Hospital, 148, Gurodong-gil, Guro-gu, Seoul 08308, Korea; jensyj85@gmail.com (S.J.); ilovewish777@naver.com (B.R.K.); clickkjl@naver.com (J.L.K.); 2 Graduate School of Medicine, College of Medicine, Korea University, Seoul 08308, Korea; qnrua10047@naver.com (B.G.K.); derrickdyblue22@gmail.com (D.Y.K.); psh3938@hanmail.net (S.H.P.); wing1278@naver.com (Y.J.N.); minjeeyoyo@nate.com (M.J.J.); katecoco@hanmail.net (H.K.Y.); leomi2614@naver.com (Y.A.J.); 3 Division of Oncology, Department of Internal Medicine, Kyung Hee University School of Medicine, 23 Kyung Hee dae-ro, Dong-dae-mun-gu, Seoul 02447, Korea; xpassion84@naver.com; 4 Department of Surgery, Korea University Guro Hospital, Korea University College of Medicine, Seoul 08308, Korea; silee@korea.ac.kr; 5 Kaiyon Bio Tech Co., Ltd, 226 Gamasan-Ro, Guro-gu, Seoul 08308, Korea; howard@kaiyonbiotech.com (H.D.K.); dkim238@kaiyonbiotech.com (D.H.K.)

**Keywords:** oxaliplatin resistance, colorectal cancer, CBD, NOS3, autophagy, mitochondrial dysfunction

## Abstract

Although oxaliplatin is an effective chemotherapeutic drug for colorectal cancer (CRC) treatment, patients often develop resistance to it. Therefore, a new strategy for CRC treatment is needed. The purpose of this study was to determine the effect of cannabidiol (CBD), one of the components of the cannabis plant, in overcoming oxaliplatin resistance in CRC cells. We established oxaliplatin-resistant cell lines, DLD-1 R and colo205 R, in CRC DLD-1 and colo205 cells. Autophagic cell death was induced when oxaliplatin-resistant cells were treated with both oxaliplatin and CBD. Additionally, phosphorylation of nitric oxide synthase 3 (NOS3) was increased in oxaliplatin-resistant cells compared to that in parent cells. Combined treatment with oxaliplatin and CBD reduced phospho-NOS3 levels and nitric oxide (NO) production and resulted in the production of reactive oxygen species (ROS) by reducing the levels of superoxide dismutase 2, an antioxidant present in the mitochondria, causing mitochondrial dysfunction. Taken together, these results suggest that elevated phosphorylation of NOS3 is essential for oxaliplatin resistance. The combination of oxaliplatin and CBD decreased NOS3 phosphorylation, which resulted in autophagy, by inducing the overproduction of ROS through mitochondrial dysfunction, thus overcoming oxaliplatin resistance.

## 1. Introduction

Colorectal cancer (CRC) is the third most common cancer in both men and women [1]. The standard treatment for CRC is chemotherapy following colon resection by surgery [2]. For chemotherapy, either FOLFOX (5-fluorouracil and leucovorin with oxaliplatin) or FOLFIRI (5-fluorouracil, leucovorin with irrinotecan) combined with bevacizumab is mainly used [3]. Despite advances in treatment strategies for CRC, the prognosis remains poor because of the high rates of metastasis [4]. Oxaliplatin is the first platinum-based drug to demonstrate clinical effectiveness against CRC, and it remains one of the most effective chemotherapeutic drug for CRC treatment along with 5-fluorouracil and leucovorin [4,5]. However, repeated and long-term administration induces drug resistance through the promotion of export from the cells and nucleotide excision repair by increasing expression of the multidrug resistance protein, glutathione, and excision repair cross-complementation group 1 [6,7]. Therefore, there is a need to explore new strategies to improve the efficiency of CRC treatment by identifying molecules and mechanisms associated with oxaliplatin resistance.

Autophagy (macroautophagy) is a process involving the lysosomal degradation of cytosolic proteins, damaged organelles, and invasive microbes in autophagosomes, which are double-membrane vesicles generated by extension of phagophores [8]. Chemotherapy acts as a stress in the cells and increases apoptosis inhibition, autophagy increase, and epithelial-mesenchymal transition (EMT)-competent phenotypes through Beclin-1, Bcl-2, mammalian target of rapamycin (mTOR), adenisine monophosphate (AMP)-activated protein kinase (AMPK), and select microRNAs. In CRC, autophagy increases EMT-competent CRC cells and acquires resistance to chemotherapeutic drugs by the TP53-dependent pathway [9].

Nitric oxide synthases (NOSs) are enzymes that catalyze the production of nitric oxide (NO) from L-arginine. NO is important for maintaining vascular tone, insulin secretion, and angiogenesis. There are three mammalian NOS isoforms: neuronal NOS (nNOS or NOS1), inducible NOS (iNOS or NOS2), and endothelial NOS (eNOS or NOS3) [10]. Although NOS expression is associated with cancer progression and metastasis, recent studies have suggested that NOS3 may inhibit tumor growth, invasion, and angiogenesis, particularly in breast cancer, and CRC [11,12,13].

Cannabidiol (CBD) is one of the major components of *Cannabis sativa* [14]. It is non-psychoactive and widely used to treat diseases, such as neurological diseases and cancer [15]. Many clinical trials for its use in glioblastoma treatment are also currently underway [16]. CBD is known to exert its antitumor effects through Noxa activation, downregulation of protein kinase B (AKT)/mTOR, and mitogen-activated protein kinase signaling [14]. However, CBD has not been studied for its potential to overcome resistance to chemotherapeutic drugs.

In this study, we investigated whether CBD overcomes oxaliplatin resistance in CRC cells, and the relationship between NOS3 downregulation and combined oxaliplatin and CBD treatment-induced autophagy. We demonstrate, for the first time, that CBD enhances oxaliplatin-mediated autophagy via NOS3-mediated mitochondrial dysfunction, suggesting that NOS3 is a potential therapeutic target for overcoming oxaliplatin resistance and that CBD may be a new therapeutic agent for CRC treatment.

## 2. Results

### 2.1. CBD and Oxaliplatin Reduce Proliferation and Induce Autophagic Death of Oxaliplatin-Resistant CRC Cells

**Figure 1:**
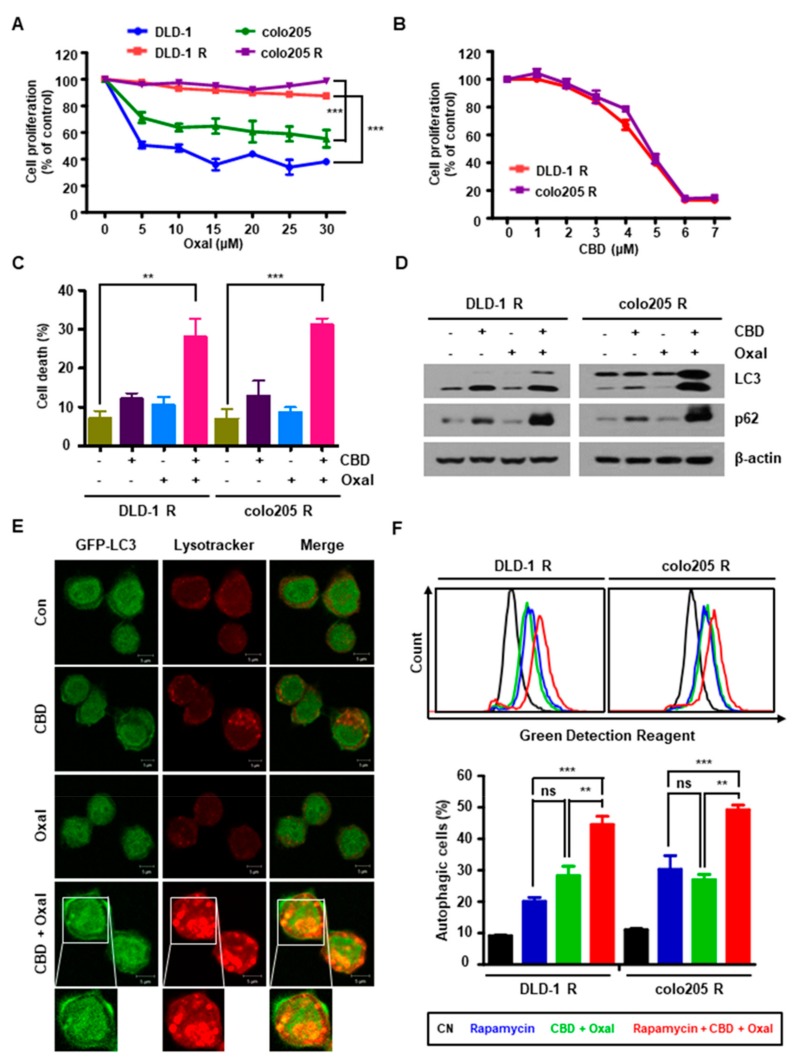
Combination of oxaliplatin and cannabidiol (CBD) reduces cell proliferation and induces autophagy in oxaliplatin-resistant colorectal cancer (CRC) cell lines. (**A**,**B**) Parent (DLD-1, colo205) and oxaliplatin-resistance CRC cell lines (DLD-1 R, colo205 R) were treated with 0–30 μM (**A**) oxaliplatin (**B**) or CBD for 24 h. Cell proliferation was measured by 4-[3-(4-lodophenyl)-2-(4-nitrophenyl)-2H-5-tetrazolio]-1,3-benzene disulfonate (WST-1) assay. *** *p* < 0.001. (**C**) After oxaliplatin and CBD treatment, viable oxaliplatin-resistant cells were identified by Trypan blue staining. ** *p* < 0.01 and *** *p* < 0.001. (**D**) Cells were treated with 4 μM CBD and 10 μM oxaliplatin for 24 h. The levels of LC3 and p62 were determined by western blotting. (**E**) The formation of green fluorescent protein (GFP)-LC3 puncta after treatment with oxaliplatin and CBD was analyzed by confocal microscope (scale bar, 5 μm). (**F**) Cells were treated with oxaliplatin and CBD for 12 h following pretreatment with 1 μM rapamycin for 1 h and then stained with an Autophagy Detection Kit for 30 min at 37 °C in the dark. Cells were harvested and analyzed by flow cytometry (upper). The graph represents the quantification of autophagic cells (lower). ** *p* < 0.01 and *** *p* < 0.001.

**Figure A1:**
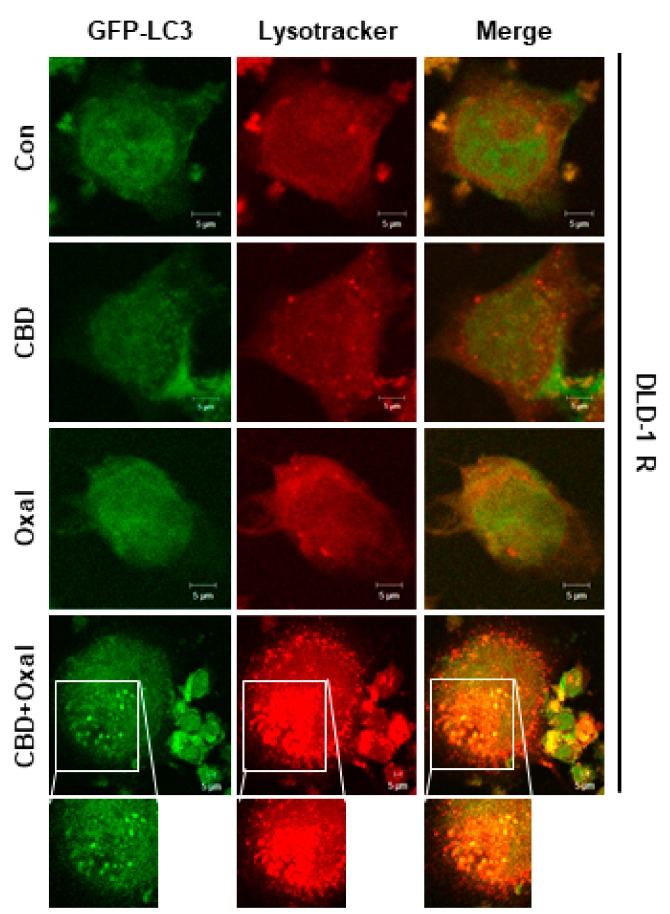
Oxaliplatin and CBD induces autophagy. The formation of GFP-LC3 puncta after treatment with oxaliplatin and CBD was analyzed by confocal microscopy in DLD-1 R cells (Scale Bar, 5 μm).

To investigate the effect of CBD on oxaliplatin resistance in CRC, we first generated the oxaliplatin-resistant cell lines, DLD-1 R and colo205 R. Oxaliplatin decreased the proliferation of the parent cells in a dose-dependent manner, but did not affect proliferation of the oxaliplatin-resistant cells (Figure 1A). However, CBD decreased the viability of oxaliplatin-resistant cells in a dose-dependent manner (Figure 1B). As shown in Figure 1C, oxaliplatin and CBD, in combination, significantly increased cell death. To determine whether the increased death of oxaliplatin and CBD-treated cells was due to autophagy, DLD-1 R and colo205 R cells were treated with oxaliplatin and CBD and autophagic changes were assessed. The combination of oxaliplatin and CBD markedly promoted microtubule-associated proteins 1A/1B light chain 3B (LC3) and p62 expression, as well as LC3 punctuation (Figure 1D,E and Figure A1), which are widely used autophagic markers [17]. Moreover, the increased autophagic activity induced by combination treatment was further enhanced by the autophagy inducer, rapamycin (Figure 1F), suggesting that combined oxaliplatin and CBD treatment induces autophagy.

### 2.2. NOS3 Is Associated with Oxaliplatin Resistance and Decreased NOS3 Activity Induces Autophagy

**Figure 2:**
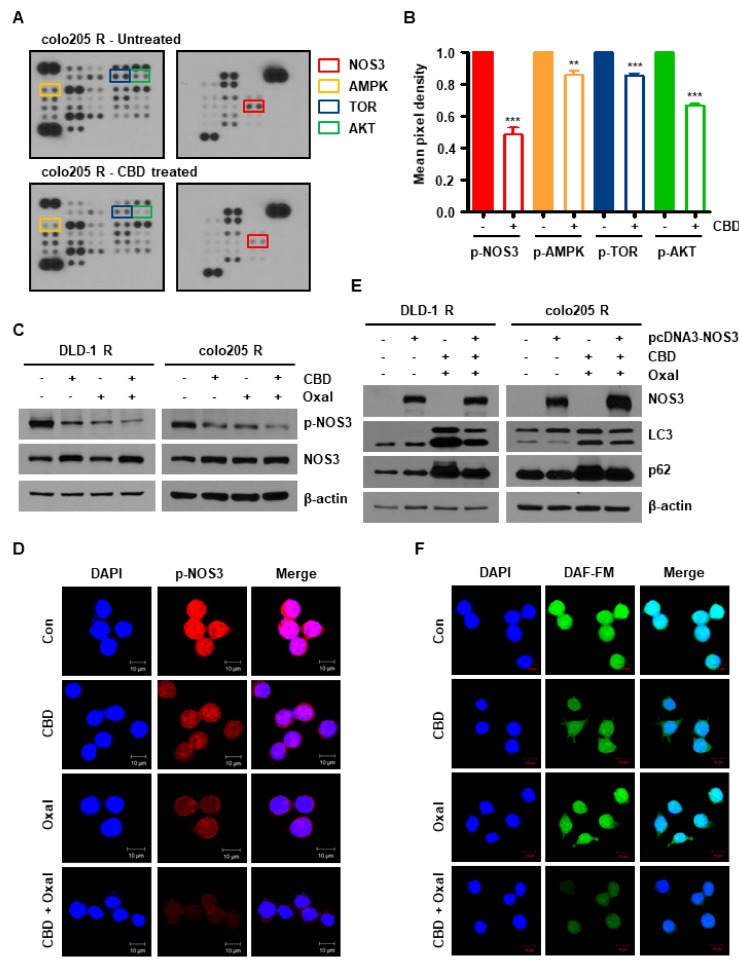
Downregulation of phospho-nitric oxide synthase 3 (NOS3) induces autophagic cell death. (**A**,**B**) colo205 R cells were treated with 4 μM CBD for 8 h. (**A**) Cell lysates were then hybridized to the Phospho-Kinase Array kit. (**B**) The graph represents the quantification of mean pixel density using the Image J program. ** *p* < 0.01 and *** *p* < 0.001. (**C**) Protein levels of NOS3 and phospho-NOS3 were determined by western blotting in oxaliplatin-resistant cells treated with oxaliplatin and CBD for 24 h. (**D**) colo205 R cells were immunostained with phopho-NOS3 following treatment with oxaliplatin and CBD. Images were captured using a confocal microscope (scale bar, 10 μm). (**E**) pcDNA3-NOS3-GFP-transfected cells were treated with oxaliplatin and CBD for 24 h. Protein levels of NOS3, phospho-NOS3, LC3, and p62 were determined by immunoblotting. (**F**) Colo205 R cells were treated with oxaliplatin and CBD. Cells were then stained with 10 μM 4-amino-5-methylamino-2’,7’-difluorescein (DAF-FM) dye for 40 min at 37 °C. Images were captured using a confocal microscope (scale bar, 10 μm).

**Figure A2:**
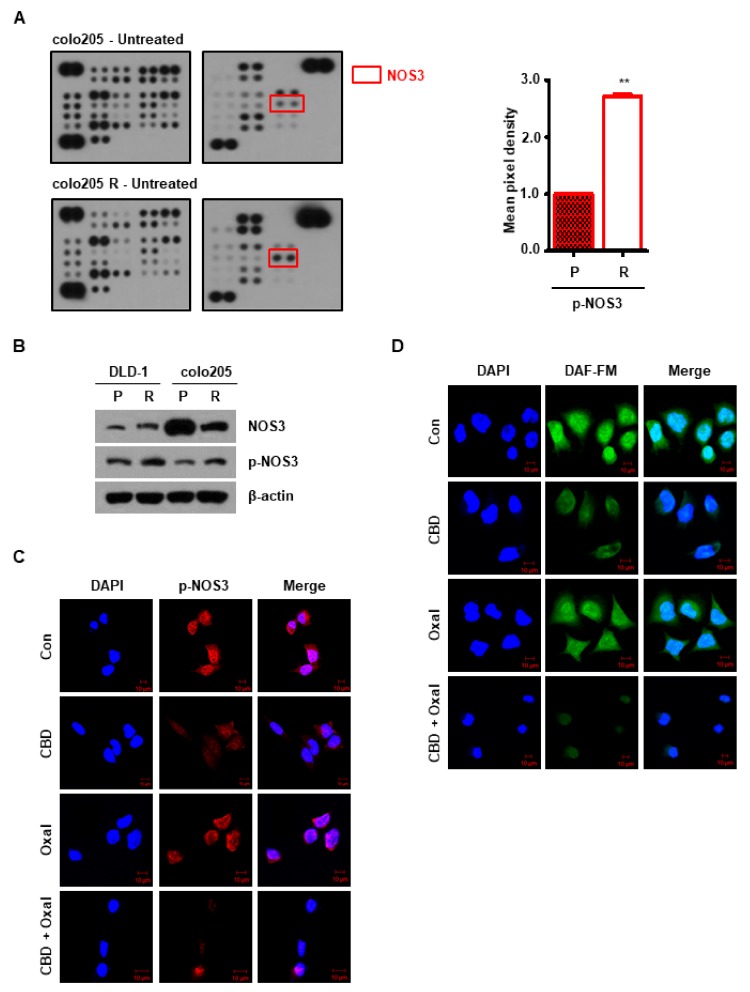
Decreased NOS3 activity induced by CBD in oxaliplatin-resistant cells inhibits NO production. (**A**) The Phospho-Kinase Array Kit was used to assay colo205 and colo205 R cells (left). The graph represents the quantification of mean pixel density of phopho-NOS3 using the Image J program (right). ** *p* < 0.01. (**B**) The levels of NOS3 and phopho-NOS proteins in parent and oxaliplatin-resistant CRC cells. (**C**,**D**) DLD-1 R cells were stained for (**C**) phopho-NOS3 or with (**D**) the DAF-FM dye following treatment with oxaliplatin and CBD. Images were captured using a confocal microscope (scale bar, 10 μm).

To identify the specific effector signaling proteins associated with oxaliplatin resistance, we detected the phosphorylation of certain proteins using a Human Phospho-Kinase Array kit. Endogenous phospho-NOS3 levels were significantly increased in oxaliplatin-resistant cells compared with the parent cells (Figure A2A,B). Additionally, CBD decreased the phosphorylation of several proteins including AKT, TOR, and AMPK associated with autophagy, and most markedly, NOS3 (Figure 2A,B). To confirm this result, we measured the levels of phospho-NOS3 using western blotting and immunofluorescence. As shown in Figure 2C,D, and Figure A2C, while total NOS3 protein levels were unchanged, combined oxaliplatin and CBD treatment attenuated the levels of phospho-NOS3. To determine the role of NOS3 downregulation in oxaliplatin and CBD-induced autophagy, oxaliplatin-resistant cells were transfected with an empty vector (pcDNA) or a NOS3-overexpressing plasmid (pcDNA3-NOS3-green fluorescent protein (GFP)). Compared to cells treated with the empty vector, NOS3 overexpression attenuated oxaliplatin and CBD-induced autophagic cell death in both cell lines, as indicated by the protein levels of LC3, and p62 (Figure 2E).

Since NOS3 regulates NO production [18], we investigated whether oxaliplatin and CBD-induced NOS3 downregulation was associated with NO generation. As shown in Figure 2F and Figure A2D, NO levels were decreased by combined oxaliplatin and CBD treatment. Taken together, these results show that NOS3 plays an important role in oxaliplatin resistance, as well as autophagic cell death induced by CBD and NO production.

### 2.3. Reduction of Antioxidant SOD2 by Oxaliplatin and CBD Induces Reactive Oxygen Species (ROS) Overproduction

**Figure 3:**
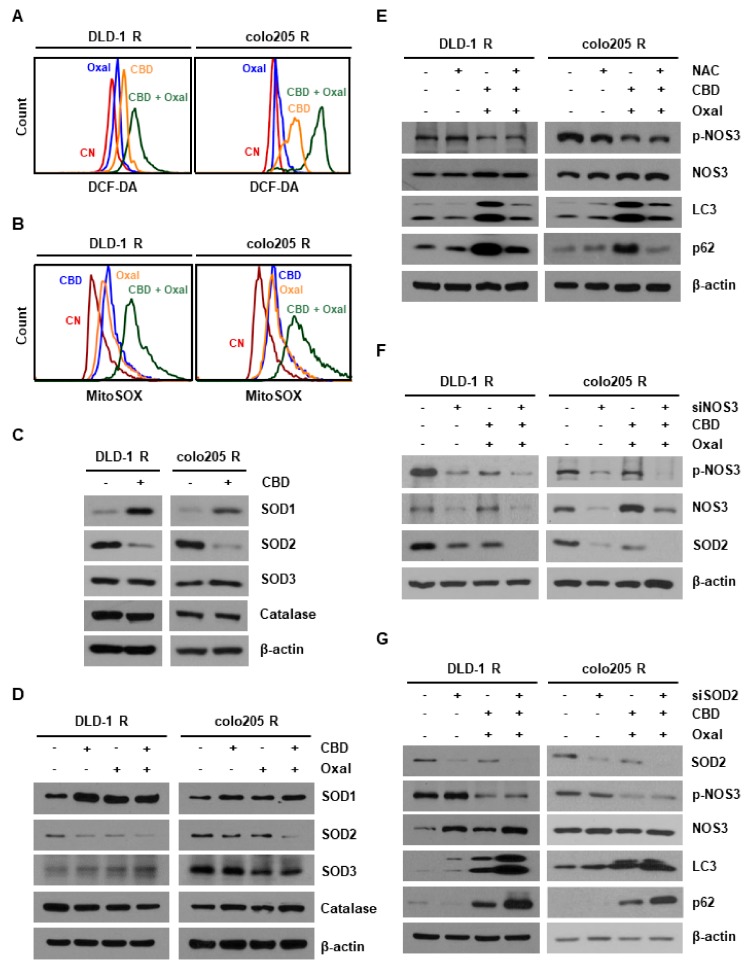
Combined treatment with oxaliplatin and CBD induces reactive oxygen species (ROS) via SOD2 inhibition. (**A**,**B**) Cells were treated with oxaliplatin and CBD for 24 h. Treated cells were then stained with (**A**) 10 μM 2’,7’-dichlorodihydrofluorescein diacetate (DCF-DA) or (**B**) 5 μM mitochondrial superoxide indicator (MitoSOX) for 10 min at 37 °C. Stained cells were harvested and analyzed by flow cytometry. (**C**) Cells were treated with 4 μM CBD for 24 h and antioxidant protein levels were then measured by western blotting. (**D**) Antioxidant protein expression was determined by western blotting in DLD-1 R and colo205 R cells treated with oxaliplatin and CBD. (**E**) Cells were pretreated with 5 μM N-acetyl cysteine (NAC) for 1 h and then treated with oxaliplatin and CBD for 24 h. Cell lysates were analyzed by western blotting. (**F**,**G**) Cells were transfected with a (**F**) *SOD2* or (**G**) *NOS3* siRNA. Transfected cells were treated with oxaliplatin and CBD for 24 h and the protein levels of SOD2, NOS3, p-NOS3, LC3, and p62 were determined by western blotting.

**Figure A3:**
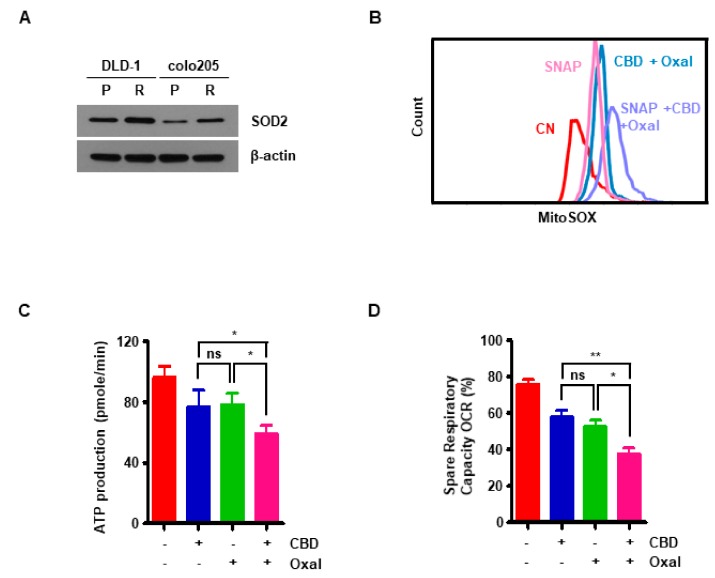
Combined treatment with oxaliplatin and CBD induces mitochondrial dysfunction by SOD2 downregulation. (**A**) The levels of NOS3 and phopho-NOS in parent and oxaliplatin-resistant CRC cells. (**B**) Cells were pretreated with 100 μM SNAP for 1 h and then treated with oxaliplatin and CBD for 24 h. Cells were then stained with MitoSOX and analyzed by flow cytometry. (**C**,**D**) colo205 R cells were treated with oxaliplatin and CBD. (**C**) ATP production and (**D**) spare respiratory capacity OCR were quantified. * *p* < 0.05 and ** *p* < 0.01.

NO and ROS are closely related [19,20]. Therefore, we examined ROS production in oxaliplatin-resistant CRC cells. As shown in Figure 3A,B, CBD and oxaliplatin enhanced intracellular ROS levels and effectively increased mitochondrial ROS production. Oxaliplatin and CBD treatment also significantly decreased the expression of superoxide dismutase 2 (SOD2), an antioxidant present in the mitochondria (Figure 3C,D). Moreover, SOD2 protein levels were increased in oxaliplatin-resistant cells compared with the parent cells (Figure A3A). We then explored the possible link between ROS overproduction and autophagy in oxaliplatin and CBD-treated cells. We found that the oxaliplatin and CBD-induced increase in autophagy was remarkably diminished by N-acetyl cysteine (NAC) pretreatment in both cell lines (Figure 3E). Moreover, to assess the relationship between NO and mitochondrial ROS, we treated cells with the NO donor, S-Nitroso-N-Acetyl-D,L-Penicillamine (SNAP), 1 h before oxaliplatin and CBD treatment. As shown in Figure A3B, oxaliplatin and CBD-induced mitochondrial ROS levels were further increased by SNAP, indicating that the reduction in NO by oxaliplatin and CBD generates mitochondrial ROS. Taken together, these results demonstrated that SOD2 is important for oxaliplatin resistance and is associated with oxaliplatin and CBD-induced autophagy through ROS overproduction.

To investigate the relationship between NOS3 and SOD2 in autophagic cell death, cells were transfected with a *SOD2* or *NOS3* siRNA. SOD2 expression was significantly decreased in *NOS3* siRNA-transfected cells (Figure 3F). However, SOD2 knockdown did not affect the levels of the NOS3 protein. Additionally, oxaliplatin and CBD-induced autophagy was partially inhibited by SOD knockdown (Figure 3G), implying that NOS3 regulates SOD2 and oxaliplatin and CBD-induced autophagy.

### 2.4. CBD and Oxaliplatin Treatment Leads to Mitochondrial Dysfunction

**Figure 4:**
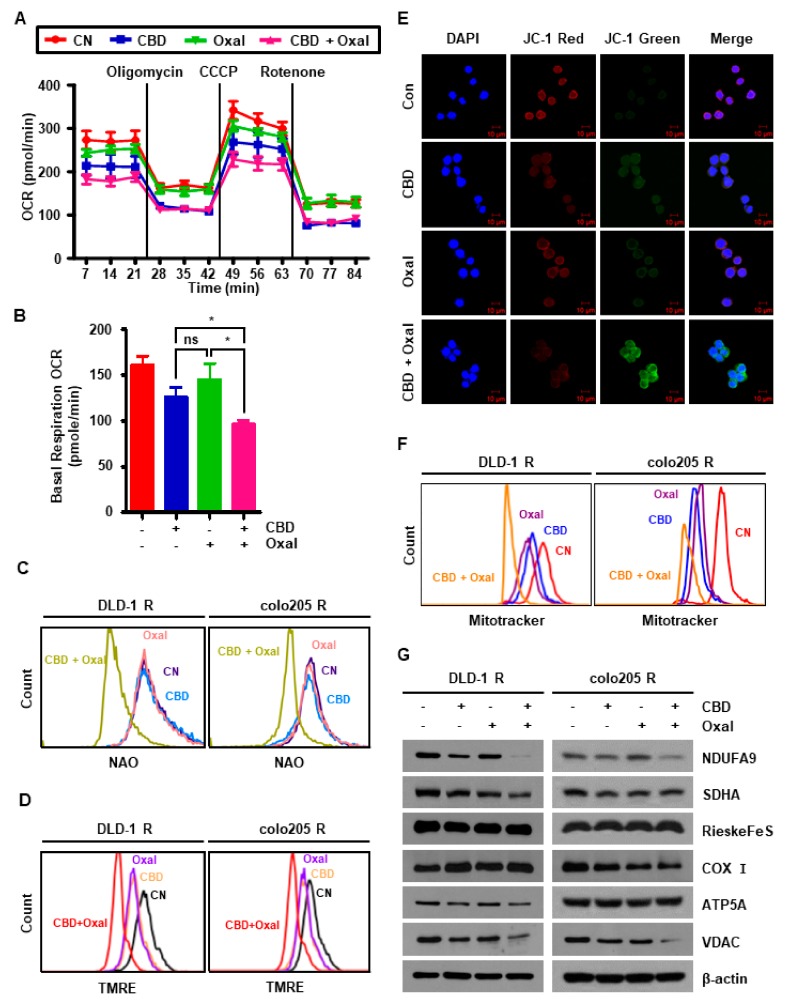
Combination of oxaliplatin and CBD leads to mitochondrial dysfunction. (**A**,**B**) Oxygen consumption rate (OCR) was measured in colo205 R cells treated with oxaliplatin and CBD using XF-24 extracellular flux analyzer. (**A**) OCR levels were detected following the addition of 2 μg/mL oligomycin, 2.5 μM carbonyl cyanide m-chlorophenyl hydrazine (CCCP), and 3 μM rotenone. (**B**) OCR was quantified during basal respiration. * *p* < 0.05. (**C–F**) Oxaliplatin and CBD-treated cells were stained with (**C**) 5 μM nonyl acridine orange (NAO), (**D**) 500 nM tetramethylrhodamine, ethyl ester, perchlorate (TMRE), (**E**) 5,5,6,6-Tetrachloro-1,1,3,3-tetraethylbenzimidazolylcarbocyanine iodide (JC-1) (scale bar, 10 μm), or (**F**) MitoTracker and analyzed by flow cytometry and immunocytochemistry. (**G**) Cells were treated with CBD and oxaliplatin for 24 h and then the protein levels of mitochondrial electron transport chain (ETC)-related proteins were measured by western blotting.

Since SOD2 and ROS mainly act on mitochondria, we measured oxygen consumption rate (OCR) to determine whether oxaliplatin and CBD affect mitochondrial function. OCR was attenuated in oxaliplatin and CBD-treated colo205 R cells (Figure 4A). In addition, the levels of OCR during basal respiration, adenosine triphosphate (ATP) production, and spare respiratory capacity OCR were significantly decreased under the same conditions (Figure 4B and Figure A3C,D). Oxaliplatin and CBD decreased the fluorescence intensity of nonyl acridine orange (NAO), which binds to the mitochondrial membrane protein, cardiolipin, and also decreased mitochondrial membrane potential (MMPs; Figure 4C–E). Moreover, we determined the number of mitochondria by staining with a MitoTracker dye and immunoblotting. As shown in Figure 4F,G, the fluorescence intensity of MitoTracker and voltage-dependent anion channel (VDAC) protein levels were diminished by oxaliplatin and CBD. Oxaliplatin and CBD-induced mitochondrial malfunction was further confirmed by measuring the expression of mitochondrial electron transport chain (ETC) proteins. Nicotinamide adenine dinucleotide dehydrogenase (NADH) dehydrogenase 1 alpha subcomplex subunit 9 (NDUFA9), one of the subunits of mitochondrial complex I, was the only ETC protein with reduced levels in cells treated with oxaliplatin and CBD (Figure 4G). Taken together, these results imply that oxaliplatin and CBD cause mitochondrial dysfunction by not only reducing the number of mitochondria, but also by inducing abnormalities in the mitochondrial membrane.

### 2.5. CBD and Oxaliplatin Inhibit Tumor Growth In Vivo by Decreasing Phospho-NOS3 and SOD2 Levels and Inducing Subsequent Autophagy

**Figure 5:**
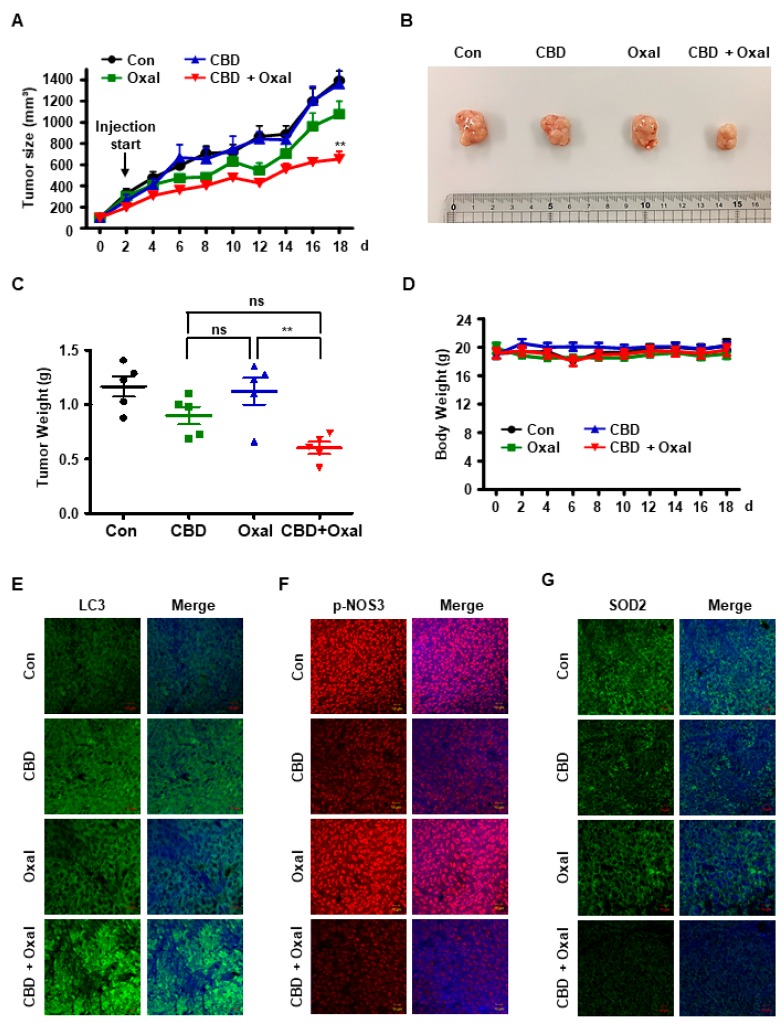
Oxaliplatin and CBD causes autophagy by downregulating phopho-NOS3 and SOD2 in vivo. (**A**–**D**) Nude BALB/c mice were subcutaneously injected with colo205 R cells and tumor growth and body weight were measured every 2 d after treatment with 5 mg/kg oxaliplatin, 10 mg/kg CBD, or their combination (*n* = 5). The line graph indicates (**A**) tumor volume (**B**) and tumor tissues were imaged using a digital camera. After the experiment was terminated, (**C**) tumor tissue and (**D**) mice were weighed. **, *p* < 0.01. (**E**–**G**) Immunohistochemical staining was performed for (**E**) LC3, (**F**) phopho-NOS3, and (**G**) SOD2 on tumor sections isolated from xenografted mice (scale bar, 10 μm).

On the basis of our in vitro results, we aimed to confirm the effect of oxaliplatin and CBD in vivo using a xenograft mouse model. Colo205 R cells were subcutaneously injected into nude BALB/c mice and the tumor size and body weight were measured every 2 d. We found that both tumor size and tumor weight were lower in the oxaliplatin and CBD-treated mice than in control mice and mice treated with either drug (Figure 5A–C). However, there were no differences in body weight among the four groups (Figure 5D). To determine whether autophagy played a role in this process, we examined LC3 levels in the tumor tissue. As shown in Figure 5E, LC3 levels were higher in tumors from oxaliplatin and CBD-treated mice than in tumors from control mice and mice treated with either drug. Next, we sought to determine whether phospho-NOS3 and SOD2 were responsible for the autophagy. Both SOD2 and phopho-NOS3 levels were lower in tumors from oxaliplatin and CBD-treated mice than in tumors from control mice (Figure 5F,G), indicating that oxaliplatin and CBD induce autophagy in vivo by downregulating phopho-NOS3 and SOD2.

## 3. Discussion

Oxaliplatin, which is commonly used for CRC treatment, binds covalently to DNA nucleobases to form guanine–guanine and guanine–adenine DNA links [4]. This abnormal binding interferes with the structure of DNA and inhibits DNA replication, repair, and transcription, triggering double-strand breaks and causing apoptosis [5]. However, drug resistance occurs in ~40% of CRCs treated with oxaliplatin [21]. Therefore, a new therapeutic strategy needs to be developed to overcome oxaliplatin resistance in CRCs. In this study, we investigated whether there is a synergistic effect when CBD is combined with oxaliplatin for CRC treatment. Additionally, we analyzed the mechanism whereby CBD overcomes oxaliplatin resistance in CRC cells and report, for the first time, that CBD downregulates NOS3 activity, resulting in mitochondrial dysfunction and finally, leading to autophagy.

Oxaliplatin resistance is acquired through a variety of mechanisms, including inefficient cellular accumulation of drugs [22], promotion of DNA repair [23], activation of anti-apoptotic pathways [24], and changes in cellular metabolism. An important finding of this study is that NOS3 is closely related to oxaliplatin resistance. NOS is an enzyme that converts L-arginine to L-citrulline to produce NO in various cell types [25]. It has been mainly studied for its function in endothelial cells. In addition, NOS has been reported to be associated with resistance to cisplatin and 5-fluorouracil through Wnt signaling and family with sequence similarity 171, member B in non-small cell lung cancer, breast cancer, and leukemia [7,26,27]; but this is a report of drug resistance regulated by NOS2. Our results showed that the activity of NOS3 was increased in oxaliplatin-resistant cells compared to that in their parent cells and this was attenuated by CBD, resulting in decreased NO production. Moreover, studies on the effects of CBD on overcoming drug resistance have rarely been performed on cancers other than glioblastoma [26,27]. Therefore, NOS3 plays an important role in acquiring resistance to oxaliplatin, and CBD can decrease NO production by reducing NOS3 activity and thus increase oxaliplatin sensitivity.

Intracellular NO produced by NOS3 directly affects mitochondrial function through oxidative stress [28,29,30]. Consistent with this, we found that CBD reduced OCR, a direct marker of mitochondrial dysfunction, and significantly decreased MMP, mitochondrial complex I activity, the number of mitochondria, and the levels of the mitochondrial membrane protein, cardiolipin. Moreover, CBD increased the levels of intracellular ROS, especially mitochondrial ROS, and decreased the protein expression of the mitochondrial antioxidant enzyme, SOD2. NOS3 knockdown further reduced SOD2 levels in cells treated with CBD and oxaliplatin, indicating that the downregulation of NOS3 induced by CBD causes mitochondrial dysfunction and ROS overproduction by SOD2 reduction.

Autophagy is known to be involved in cell survival by regulating intracellular homeostasis in response to stress [31]. However, in recent decades, autophagy has also been reported to be associated with cell death [32]. Previous studies have suggested that autophagy promotes the resistance of cancer cells to chemotherapy [33,34]. In contrast to those of previous studies, our results showed that the induction of autophagy by CBD increased the response of cells to oxaliplatin. The reason for this discrepancy is unclear, but it may be due to differences in the mechanism of action of CBD or different cellular responses to oxaliplatin compared with other chemotherapy drugs.

Moreover, one of the serious side effects of oxaliplatin is neurotoxicity induced by the pro-inflammatory cytokines, interleukin 6, tumor necrosis factor alpha, and cyclooxygenase 2 [35,36]. CBD enhances antioxidant activity and inhibits the secretion of pro-inflammatory cytokines and thus, possibly acts as a neuroprotectant [37,38,39]. Therefore, CBD may attenuate the neurotoxic side effects of oxaliplatin.

## 4. Materials and Methods

### 4.1. Cell Lines and Cell Culture

Human CRC DLD-1 and colo205 cells were purchased from the American Type Culture Collection (Manassas, VA, USA). Oxaliplatin-resistant DLD-1 (DLD-1 R) and colo205 (colo205 R) cells were developed by long-term exposure to oxaliplatin, with stepwise increases in concentration. All cells were cultured in RPMI 1640 medium (Gibco, Grand Island, NY, USA) supplemented with 10% fetal bovine serum and 100 mg/mL penicillin and streptomycin in a 5% CO_2_ atmosphere incubator at 37 °C.

### 4.2. Establishment of Oxaliplatin-Resistant Cell Lines

Resistance to oxaliplatin was induced by exposing cells to increased concentrations of oxaliplatin (1–20 µM) for 6 months. Initially, 1 µM oxaliplatin was added to the medium, and surviving cells were cultured every 4–5 days. Oxaliplatin dosage was gradually increased when the effect of the drug was considered insignificant. In both cell lines (colo205 R, DLD-1 R), we confirmed the resistance to oxaliplatin in comparison to the parent cells (colo205, DLD-1).

### 4.3. Reagents and Antibodies

CBD, oxaliplatin, 4-amino-5-methylamino-2’,7’-difluorescein (DAF-FM), SNAP, and NAC were purchased from Sigma Aldrich (St. Louis, MO, USA). 2’,7’-dichlorodihydrofluorescein diacetate (DCF-DA), mitochondrial superoxide indicator (MitoSOX), tetramethylrhodamine, ethyl ester, perchlorate (TMRE), 5,5,6,6-Tetrachloro-1,1,3,3-tetraethylbenzimidazolylcarbocyanine iodide (JC-1), MitoTracker, and NAO were purchased from Thermo Fisher Scientific (Waltham, MA, USA). Specific antibodies against LC3A/B, NOS3, SOD1, and VDAC were purchased from Cell Signaling Technology (Danvers, MA, USA). Antibodies against SOD3, succinate dehydrogenase complex flavoprotein subunit A (SDHA), RieskeFeS, COX I, and ATP synthase F1 subunit alpha (ATP5A) were purchased from Santa Cruz Biotechnology (Dallas, TX, USA). Antibodies against phospho-NOS3 (S1177), p62, NDUFA9 were purchased from Abcam (Cambridge, UK). The anti-SOD2 antibody was purchased from Enzo Life Sciences (Farmingdale, NY, USA) and the anti-β-actin antibody was purchased from Sigma Aldrich. Horseradish peroxidase-conjugated anti-mouse IgG was purchased from Santa Cruz Biotechnology and anti-rabbit IgG was from Cell Signaling Technology.

### 4.4. Cell Proliferation Assay

Cells were seeded onto 96-well plates at a density of 1 × 10^4^ cells/well and incubated overnight. They were then treated with CBD or oxaliplatin for 24 h. Subsequently, WST solution was added to each well for 2 h. The absorbance at 450 nm was then measured using a microplate reader (SPECTRA190; Molecular Devices, Sunnydale, CA, USA).

### 4.5. Immunoblotting

Immunoblotting was performed as previously described [40].

### 4.6. Green Fluorescent Protein (GFP)-LC3 Puncta

Cells were infected with recombinant adenoviruses expressing GFP-LC3 (a gift from Professor Chang Kyu Lim, Chungnam National University, Daejeon, Korea). Infected cells were then incubated with CBD and oxaliplatin for 6 h and stained with 5 μM lysotracker dye for 30 min at 37 °C. The cells were observed under a confocal microscope (Carl Zeiss, Oberkochen, Germany).

### 4.7. Autophagic Activity

The Autophagy Detection Kit (Abcam) measures autophagic activity in living cells using a fluorescent detection reagent. Cells were seeded in culture dishes, incubated overnight. Following incubation, they were washed with phosphate-buffered saline (PBS) and pretreated with 1 μM rapamycin (an autophagy inducer) in serum-free RPMI medium for 1 h at 37 °C. Cells were then treated with CBD and oxaliplatin for 12 h and subsequently, incubated with 1 μL of Green Detection Reagent for 30 min at 37 °C in the dark. Cells were harvested using trypsin- ethylenediaminetetraacetic acid (EDTA) and resuspended with 500 μL of 1× assay buffer. Cells were analyzed on the FL-1 fluorescence channel of a flow cytometer.

### 4.8. Analysis of Cell Death

After treatment with oxaliplatin and CBD, cells were harvested using trypsin-EDTA and stained with Trypan blue. Triplicate wells of viable cells were counted using a hemocytometer.

### 4.9. Human Phospho-Kinase Array

Cell lysates were assayed using a Proteome Profiler Human Phospho-Kinase Array kit (R&D Systems, Minneapolis, MN, USA), according to the manufacturer’s instructions.

### 4.10. Immunofluorescence Staining

After CBD and oxaliplatin treatment, cells were fixed, permeabilized, blocked, and incubated with primary antibodies. Bound primary antibodies were visualized using an Alexa Fluor-594-conjugated secondary antibody (Molecular Probes, Eugene, OR, USA) and cells were stained with 4’,6-diamidino-2-phenylindole (Invitrogen, CA, USA). Finally, cells were mounted and imaged using a confocal microscope.

### 4.11. Measurement of ROS

DLD-1 R and colo205 R cells were treated with CBD and oxaliplatin for 24 h and subsequently, incubated with 10 μM DCF-DA and 5 μM MitoSOX dye for 30 min at 37 °C. Cells were then harvested and the fluorescence intensity, indicating the level of ROS, was quantitated by flow cytometry.

### 4.12. Transfection

Small interfering RNAs (siRNAs) targeting *NOS3* and *SOD2* were purchased from Santa Cruz Biotechnology. pcDNA3-NOS3-GFP and pBI-EGFP-SOD2 plasmids were purchased from Addgene (Watertown, MA, USA). Cells were transfected with siRNAs or plasmids using Lipofectamine RNA iMAX or Lipofectamine 2000 reagent, respectively (Invitrogen, Carlsbad, CA, USA).

### 4.13. OCR

OCR was measured in DLD-1 R and colo205 R cell lines using the Seahorse XF-24 extracellular flux analyzer (Seahorse Biosciences, MA, USA). Cells were seeded in Seahorse XF-24 cell culture microplates at a density of 2.5 × 10^4^ cells/well and cultured for 24 h. Cells were then washed with PBS and incubated in serum-free RPMI, with 4 μM CBD and 10 μM oxaliplatin, for 12 h. Finally, cells were treated with 2 μg/mL oligomycin (ATP synthase inhibitor), 2.5 μM carbonyl cyanide *m*-chlorophenyl hydrazine (CCCP), and 3 μM rotenone (mitochondrial complex I inhibitor).

### 4.14. Determination of Mitochondrial Function

Cells were treated with 500 nM TMRE, 5 μM MitoTracker, and 5 μM NAO reagent before treatment with CBD and oxaliplatin. Cells were then analyzed by flow cytometry. Cells were treated with 5 μM JC-1 the following day after CBD and oxaliplatin treatment. They were then mounted and visualized under a confocal microscope.

### 4.15. In Vivo Tumor Xenograft Model

Animal experiments were performed according to the Guidelines and Regulations for the Care and Use of the Korea University Institutional Animal Care and Use Committee (KOREA-2018-0083). Four-week-old female BALB/c nude mice were acclimated for 1 week prior to the study and were provided free access to food and water. colo205 R cells (1 × 10^7^) in 100 μL of PBS were subcutaneously injected into 4-week-old female BALB/c nude mice. Tumor size and body weight were measured every 2 days. CBD and oxaliplatin were intraperitoneally injected at the same time. Tumor volume was calculated using the formula, 0.5 × length × (width)^2^. Six mice were included in each treatment group.

### 4.16. Immunohistochemistry (IHC)

IHC was carried out as previously described [41]. The tissue was observed by confocal microscopy (ZEISS-LSM 700, ZEISS, Oberkochen, Germany).

### 4.17. Statistical Analysis

All experiments were performed in triplicate and each yielded similar result. Results are presented as an average of three independent experiments. Statistical analysis was performed using Prism 6 software (GraphPad, San Diego, CA, USA). The results are expressed as the mean of arbitrary values ± SEM. All results were evaluated using an unpaired Student’s *t* test, in which a *p*-value of less than 0.05 was considered significant (*, **, and *** indicates *p* < 0.05, *p* < 0.01, and *p* < 0.001, respectively).

## 5. Conclusions

To summarize, NOS3 plays an important role in oxaliplatin resistance and CBD overcomes NOS-induced oxaliplatin resistance by inducing autophagy. This enhanced autophagy is triggered by mitochondrial dysfunction through a reduction in SOD2 expression. Additionally, because CBD can attenuate the side effects of oxaliplatin, our findings suggest that CBD has potential as a new therapeutic agent to combine with other modalities and drugs to treat CRC.
